# Optic nerve head vascular variations in pseudoexfoliative and primary open-angle glaucoma

**DOI:** 10.5935/0004-2749.2021-0420

**Published:** 2023

**Authors:** Sinem Karabulut, Ahmet Kaderli, Müjdat Karabulut, Sabahattin Sül, Aylin Karalezli

**Affiliations:** 1 Department of Ophthalmology, Mugla Sıtkı Kocman University Medical School, Mugla, Turkey

**Keywords:** Optic disk, Glaucoma, open-angle, Nerve fibers, Retinal vessels, Tomography, optical coherence, Computed tomography, Disco óptico, Glaucoma de ângulo aberto, Fibras nervosas, Vasos retinianos, Tomografia de coerência óptica, Angiografia por tomografia computadorizada

## Abstract

**Purpose:**

The purpose of this study was to assess the optic nerve head microvascular changes in pseudoexfoliative and primary open-angle glaucoma and define the relationship between vessel density and retinal nerve fiber layer thickness.

**Methods:**

This observational cross-sectional study assessed 72 eyes with primary open-angle glaucoma, 41 eyes with pseudoexfoliative glaucoma, and 60 healthy eyes. On the basis of optic nerve head-centered, 4.5 mm × 4.5 mm scan size images, we evaluated the vessel density, as well as the peripapillary sector, inside disk, and all sectoral quadrants.

**Results:**

Both glaucoma Groups had lower vessel density in all regions compared with the healthy Group (p<0.05 for all variables). Vessel densities of the nasal inferior, inferior nasal, and inferior temporal sectors in both glaucoma Groups showed similar results (p=0.157, p=0.128, p=0.143, respectively). Eyes with pseudoexfoliative glaucoma had significantly lower vessel densities than eyes with primary open-angle glaucoma in all other regions (p<0.05 for all variables). For both glaucoma Groups, the average retinal nerve fiber layer thickness positively correlated with vessel density in all peripapillary sectors (p<0.05 for all variables).

**Conclusions:**

Reduction in vessel density correlated with the thinning of retinal nerve fiber layer in both glaucoma Groups. Decreased vessel density in the optic nerve head can be used to demonstrate the microvascular pathologies and possible ischemic changes that lead to faster progression and worse prognosis in pseudoexfoliative glaucoma.

## Abstract (pt)

**Objetivo:**

Atribuir variações microvasculares à cabeça do nervo óptico no glaucoma pseudoesfoliativo e primário de ângulo aberto, e definir a relação entre a densidade dos vasos e a espessura da camada de fibras nervosas da retina.

**Métodos:**

Este estudo foi projetado como observacional e transversal. Foram incluídos 72 olhos com glaucoma primário de ângulo aberto, 41 olhos com glaucoma pseudoesfoliativo e 60 olhos saudáveis. Foram obtidas imagens do nervo óptico centralizadas na cabeça do nervo com 4,5 × 4,5 mm de tamanho de varredura. A densidade vascular foi avaliada em toda a imagem, na área peripapilar, dentro do disco óptico e em todos os quadrantes setoriais.

**Resultados:**

Em todas as regiões, a densidade vascular foi menor em ambos os grupos com glaucoma que nos olhos saudáveis (p<0,05 para todas as variáveis). Em ambos os grupos com glaucoma, a densidade vascular mostrou resultados semelhantes nos setores nasal inferior, inferior nasal e temporal inferior (respectivamente, p=0,157, p=0,128 e p=0,143). Os olhos com glaucoma pseudoesfoliativo mostraram densidade vascular acentuadamente menor que nos olhos com glaucoma primário de ângulo aberto em todas as outras regiões (p<0,05). A espessura média da camada de fibras nervosas da retina demonstrou uma correlação positiva com a densidade vascular em todos os setores peripapilares em ambos os grupos com glaucoma (p<0,05 para todas as variáveis).

**Conclusões:**

A redução da densidade vascular foi correlacionada a uma redução da espessura da camada de fibras nervosas da retina em ambos os grupos com glaucoma. A densidade vascular reduzida na cabeça do nervo óptico poderia ser usada para provar patologias microvasculares e possíveis alterações isquêmicas responsáveis por uma evolução mais rápida e um prognóstico pior no glaucoma pseudoesfoliativo.

## INTRODUCTION

Primary open-angle glaucoma (POAG) is a chronic and progressive ocular disorder that causes irreversible damage to retinal ganglion cells and axons. Intraocular pressure (IOP) is a well-known risk and prognostic factor for POAG. Currently, ischemic vascular changes have been linked to glaucoma and its poor progression^(1)^.

On the other hand, pseudoexfoliative glaucoma (PXG) is the most prevalent and detectable etiology of open-angle glaucoma, usually having a worse prognosis and quicker progression than POAG. In addition to elevated IOP, ocular vascular pathologies and ischemic changes have been linked to the fast progression in PXG^(2)^.

Optical coherence tomography angiography (OCT-A) is a noninvasive technique for imaging the retinal vasculature using the motion contrast process^(3)^. The split-spectrum amplitude-decorrelation angiography algorithm analyzes the decorrelation signal within subsequent sweeps in OCT-A to recreate a blood flow scheme. Some studies that used OCT-A have reported a reduction in the vessel density (VD) of the optic nerve head in eyes with POAG^(4,5,6)^. However, optic nerve head microvascular changes in POAG and PXG have not been widely compared and evaluated.

This study aimed to assess the optic nerve head microvascular changes in eyes with PXG and eyes with POAG and define the relationship between VD and retinal nerve fiber layer (RNFL) thickness.

## METHODS

This observational cross-sectional study assessed 72 eyes of 40 patients with POAG, 41 eyes of 25 patients with PXG, and 60 healthy eyes of 30 age- and sex-matched subjects. Control subjects applied to our clinic for routine ophthalmologic examination and had no glaucoma finding in the ocular investigation, visual field (VF) test, and RNFL thickness analysis.

**Figure 1:**
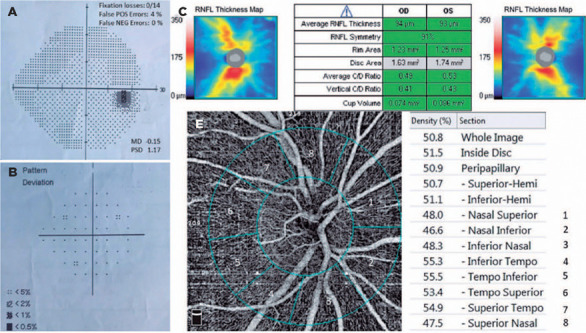
Images of a control eye’s VF (A, B), RNFL thickness map (C), and OCT-A (E). Peripapillary region (E) was automatically divided into eight sectors. Vessel density in each sector was calculated and shown in a separate cell.

We had all participants undergo a complete ophthalmologic checkup, including anterior-posterior segment examination, RNFL thickness analysis (Figure 1) with optical coherence tomography (OCT) (Zeiss Cirrus HD-OCT, Zeiss Meditec. Inc, Germany), best-corrected visual acuity test, and gonioscopic evaluation. We measured the IOP using Goldmann applanation tonometry. We conducted the VF test using Humphrey Field Analyzer (Carl Zeiss Meditec740i, Inc., Dublin, CA, USA) Swedish Interactive Thresholding Algorithm (SITA) 24-2 strategy (Figure 1).

OCT-A= optic coherence tomography angiography; RNFL= retinal nerve fiber layer; VF= visual field.

We excluded unreliable VF test results with >15% false positives, >15% false negatives, or >20% fixation losses. We also excluded patients with retinal vascular diseases (i.e., any stage of diabetic and hypertensive retinopathy, senile maculopathy, vasculitis, and uveitis), any nystagmus and amblyopia type, a history of previous ocular surgery (except uncomplicated cataract surgery within 12 months), fixation inabilities, nonglaucomatous optic neuropathies, any media opacity limiting acceptable image quality, myopia >5 diopters (D), hypermetropia and astigmatism >3 D, IOP >25 mmHg during measurement, or axial length >26 mm and <20 mm. We also excluded active smokers. In addition, we excluded images with artifacts and signal strength index <60.

We graded glaucoma for mean deviation (MD) in the VF test. We defined those with MDs greater than −6 dB, between −6 and −12 dB, and less than −12 dB as mild, moderate, and severe glaucoma, respectively^(7)^.

Both POAG and PXG were diagnosed through ocular examination (notching or rim thinning on optic nerve head, open-angle on gonioscopy), OCT finding (RNFL thinning outside the 95% confidence interval [CI]), and detection of glaucomatous VF defects (nasal step, paracentral, arcuate, or altitudinal scotomas, and temporal wedge defects). Moreover, PXG was diagnosed in the presence of exfoliation material as shown in biomicroscopic examination.

**Figure 2:**
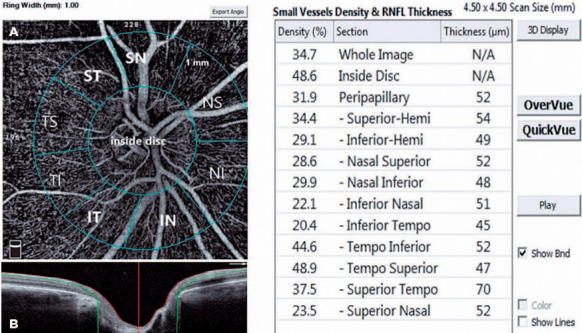
An OCT-A image of the optic nerve head in a 4.5 × 4.5 mm scan size shows the inside disk and peripapillary sectors. The peripapillary region is shown as a 1-mm wide round annulus stretching from the optic disk edge and is divided into sectors (A). The vessel densities of superficial layers from the RPC segment extending from the inner limiting membrane to the RNFL (B).

A single person generated the optic nerve head images using an RTVue-XR Avanti (Optovue, Inc., Fremont, CA, USA) on a 4.5 × 4.5 mm scan size positioned on the papilla (Figure 2). This person was a nurse with 9 years of experience in ophthalmology and did not know the study’s name, purpose, and design. The device has an angiographic program that analyzes the functional vascular anatomy of a specimen. An author who was masked to the Groups analyzed the OCT-A results and data. We recorded the VD (whole, peripapillary, and other five sectors) parameters and calculated these using the software embedded in the devices (Figure 2).

IN= inferior nasal; IT= inferior temporal; NI= nasal inferior; NT= nasal temporal; OCT-A= optic coherence tomography angiography; RNFL= retinal nerve fiber layer; SN= superior nasal; ST= superior temporal.

We used the Statistical Package for the Social Sciences version 21.0.0.0 (IBM Corporation and other(s) 1989, 2012) for data analysis. We tested the normal distribution of variables using Shapiro-Wilk W tests. In addition, we represented the demographic data as the mean ± standard deviation (SD) for continuous variables and frequencies (percentages) for categorical variables. We then calculated the mean and 95% CI for other customarily distributed variables. We used the χ^2^ test to compare categorical variables. We applied analysis of variance (ANOVA) with post hoc Tukey’s test to compare numeric parameters among POAG, PXG, and control Groups. We also used Pearson’s correlation coefficient to determine correlations. We considered a p-value <0.05 as the cutoff for significance. Finally, we performed power analysis using GPower 3.1 software.

This study was carried out in the Glaucoma Unit of the Department of Ophthalmology, Mugla Sıtkı Kocman University Hospital, Mugla, Turkey, following the Koczan Declaration of Helsinki. All patients provided informed consent. We obtained ethical approval from the Mugla Sıtkı Kocman University Clinical Research Ethics Committee (date and decision number: 30/01/2020; 02/V).

## RESULTS

The POAG Group comprised 34 (47.2%) eyes with mild glaucoma, 24 (33.3%) moderate, and 14 (19.4%) severe glaucoma. On the other hand, the PXG Group comprised 19 (46.3%) eyes with mild glaucoma, 12 (29.3%) moderate, and 10 (24.4%) severe glaucoma. The distribution of glaucoma Groups in terms of disease severity showed similar results for mild, moderate, and severe glaucoma (p=0.435, p=0.261, p=0.173; respectively). The mean RNFL thickness, vertical cup-disk ratios, and VF-MD values for the POAG and the PXG Groups were similar but significantly differed in the control Group (p<0.001 for all parameters). Age and gender were similar for all Groups (p=0.589, p=0.164; respectively). In addition, the mean IOP and central corneal thickness (CCT) were also similar for all Groups (p=0.284, p=0.326; respectively). The POAG and PXG Groups used an equal number of topical antiglaucoma medications (2.28 ± 0.8 for the POAG Group and 2.51 ± 1.22 for the PXG Group) (Table 1).

**Table 1:** Clinical and demographic findings for all groups

	POAG (n=72)	PXG (n=41)	Control (n=60)	p-value	Post hoc
	n (%)				
Glaucoma grade					
Mild	47.2/34	46.3/19	-	.435	
Moderate	33.3/24	29.3/12	-	.261	
Severe	19.4/14	24.4/10	-	.173	
**Female/Male**					
Gender	19/21	12/13	17/13	.164	A = B = C
**Mean ± SD**					
Age (years)	66.3 ± 7.5	67.9 ± 5.5	67.3 ± 5.2	.589	A = B = C
CCT (µm)	539.5 ± 31.9	542.7 ± 28.9	554.0 ± 25.3	.326	A = B = C
IOP (mmHg)	14.61 ± 3.22	15.12 ± 2.41	14.2 ± 2.7	.284	A = B = C
Medications (*n*)	2.28 ± 0.8	2.51 ± 1.22	-	.04	A = B > C
aRNFL (µm)	86.93 ± 17.38	79.93 ± 19.10	102.80 ± 6.3	<.001	A = B < C
C/D ratio (Vertical)	0.70 ± 0.23	0.62 ± 0.27	0.39 ± 0.16	<.001	A = B > C
MD (dB)	-8.19 ± 5.99	-9.66 ± 8.26	0.05 ± 0.7	<.001	A = B < C

Variables by subject; results are shown in mean ± standard deviation. Categorical variables were compared using the χ^2^ test. Other demographic parameters were compared using ANOVA and post hoc Tukey’s honest signifiicant difference test. Similar and different groups were indicated using equal (=), greater than (>), and less than (<) symbols. A linear mixed model was used to compare ocular parameters. Values with statistical significance are shown in bold. A, B, and C are POAG, PXG, and control groups.

aRNFL= average retinal nerve fiber layer; CCT= central corneal thickness; C/D= cup-disk ratio; dB= decibels; F= female; IOP= intraocular pressure; M= male; MD= mean deviation; *n*, number; POAG= primary open-angle glaucoma; PXG= pseudoexfoliation glaucoma; SD= standard deviation.

**Figure 3:**
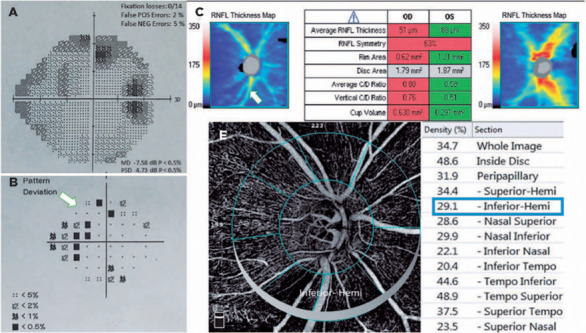
Images of VF defects (A, B), RNFL thinning (C), and vessel density reduction in an eye with PXG. The RNFL thickness map (C) shows the thinning of the nerve fiber layer, particularly in the inferotemporal segment (arrow). The pattern deviation plot (B) indicates scotomas more prominent in the superior hemifield (arrow) associated with inferotemporal RNFL defect. Vessel density reduction in inferior-Hemi (E) is more significant than that in superior-Hemi compared with the control eyes, possibly originating from the prominent thinning of inferotemporal RNFL.

**Figure 4:**
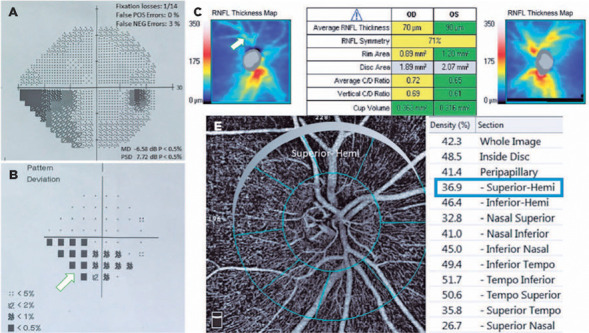
Images of VF defects (A, B), RNFL thinning (C), and vessel density reduction in an eye with POAG. The RNFL thickness map (C) shows a superior nerve fiber layer defect (arrow). The pattern deviation plot (B) indicates inferior arcuate scotoma (arrow). Vessel density reduction in superior-Hemi (E) is more significant than that in inferior-Hemi compared with the control eyes, possibly related to a superior RNFL defect.

The POAG and PXG Groups had lower mean VD in all regions compared with the control Group (p<0.05 for all areas). Figures 3 and 4 show the VF defects, RNFL thinning, and VD reduction in eyes with PXG and POAG. Although the PXG Group had lower mean VD in the nasal inferior, inferior nasal, and inferior temporal sectors than the POAG Group, these differences were not significant (p=0.157, p=0.128, p=0.143; respectively). The PXG Group showed significantly lower mean VD in the whole image as well as the peripapillary, inside disk, and other five sectors than the POAG Group (p<0.05 for all areas) (Table 2). The average RNFL thickness positively correlated with VD in all peripapillary sectors in the POAG and PXG Groups compared with that in the control Group (p<0.05 for all sectors) (Table 3). We applied post hoc power calculations, and the sample size provided 0.998 power and 1.574 effect size at α error probability level of 0.05.

PXG= pseudoexfoliation glaucoma; RNFL= retinal nerve fiber layer; VF= visual field.

POAG= primary open-angle glaucoma; RNFL= retinal nerve fiber layer; VF= visual field.

**Table 2:** Sectoral comparison of the optic nerve head vessel density in POAG, PXG, and control Groups

Density (%)	POAG (n=72)	PXG (n=41)	Control (n=60)	p^A^	p^B^	p^C^
	Mean ± SD					
Whole image	44.10 ± 7.07	39.85 ± 8.14	49.4 ± 2.7	.004	.001	<.001
Inside disk	49.81 ± 7.62	46.51 ± 7.16	53.4 ± 3.9	.035	.04	<.001
Peripapillary	45.32 ± 8.24	40.03 ± 9.66	51.3 ± 3.4	.002	.002	<.001
Nasal superior	43.99 ± 8.38	37.52 ± 10.07	48.5 ± 4.1	<.001	.03	<.001
Nasal inferior	41.22 ± 8.11	38.51 ± 8.82	47.0 ± 4.3	.157	.001	<.001
Inferior nasal	41.36 ± 12.54	37.17 ± 11.86	49.1 ± 6.5	.128	.004	<.001
Inferior temporal	47.27 ± 13.35	42.75 ± 14.12	56.9 ± 3.5	.143	.001	<.001
Temporal inferior	48.34 ± 7.94	42.79 ± 9.37	56.1 ± 4.8	.001	<.001	<.001
Temporal superior	52.27 ± 6.57	45.88 ± 8.89	59.0 ± 3.3	<.001	<.001	<.001
Superior temporal	47.25 ± 11.40	41.69 ± 13.25	56.2 ± 7.4	.026	.001	<.001
Superior nasal	42.20 ± 11.15	35.88 ± 12.81	49.3 ± 7.6	.007	.006	<.001

In the POAG and PXG Groups, vessel densities in all sectors were significantly reduced compared with those in the control Group. In the PXG Group, vessel density was significantly decreased in all sectors except in the nasal inferior, inferior nasal, and inferior temporal sectors compared with that in the POAG Group. Values with statistical significance are shown in bold. POAG= primary open-angle glaucoma; PXG= pseudoexfoliation glaucoma; *p*^A^= POAG vs. PXG; *p*^B^= POAG vs. control; *p*^C^= PXG vs. control; SD= standard deviation; *n*= number.

**Table 3:** The relation between average RNFL and peripapillary vessel density in all groups

Average RNFL						
Density (%)	POAG (n=72)		PXG (n=41)		Control (n=60)	
	r	p-value	r	p-value	r	p-value
Peripapillary	0.738	<0.001	0.874	<0.001	0.226	0.231
Nasal superior	0.680	<0.001	0.776	<0.001	0.075	0.694
Nasal inferior	0.692	<0.001	0.797	<0.001	0.056	0.769
Inferior nasal	0.648	<0.001	0.826	<0.001	0.027	0.889
Inferior temporal	0.587	<0.001	0.742	<0.001	0.053	0.780
Temporal inferior	0.493	<0.001	0.695	<0.001	0.047	0.803
Temporal superior	0.442	<0.001	0.766	<0.001	0.182	0.336
Superior temporal	0.659	<0.001	0.819	<0.001	0.146	0.441
Superior nasal	0.685	<0.001	0.791	<0.001	0.139	0.465

Compared with that in the control Group, the average RNFL positively correlated with all sectoral peripapillary vessel density in the POAG and PXG Groups. Values with statistical significance are shown in bold.

POAG= primary open-angle glaucoma; PXG= pseudoexfoliation glaucoma; r= Pearson correlation coefficient; RNFL= retinal nerve fiber layer.

## DISCUSSION

In ocular pseudoexfoliation syndrome, accruing fibrillar materials in the extracellular matrix can lead to PXG, zonular weakness, and iris atrophy. However, previous studies have found that the effects of these materials on vascular structures and functions cannot be detected directly^(8,9,10)^.

Vascular changes and remodeling have been shown to have an essential role in the physiopathology of glaucoma.

Many studies using color Doppler imaging, fluorescein angiography, or confocal scanning laser ophthalmoscopic angiography had detected a decrease in peripapillary blood flow, proving the relation between glaucoma and vascular pathology^(1,11,12,13,14)^. However, few evidences about the vascular factors on the pathophysiology of pseudoexfoliation-related glaucoma had been found.

In a recent study, Suwan et al.^(15)^ reported that eyes with POAG and PXG had lower peripapillary capillary density compared with healthy eyes and that eyes with PXG had lower peripapillary capillary density than eyes with POAG, which are congruent with our outcomes. Furthermore, our study evaluated all quadrants separately with a much larger sample size than theirs.

Previous studies had associated ocular involvement of systemic exfoliation syndrome with anterior segment hypoxia and decreased ocular-retrobulbar blood flow^(16,17)^. In addition, a decreased end-diastolic blood flow rate in the posterior ciliary arteries had been linked to perfusion disorder in exfoliation syndrome^(17)^. Moreover, the reduced VD in the optic nerve head could explain the relationship between vascular impairment and faster progression, deterioration, and unsatisfactory response to IOP control in eyes with PXG.

Park et al.^(18)^ compared the peripapillary VD of the optic nerve head and RNFL thickness in PXG and POAG eyes and found that PXG eyes had significantly lower peripapillary VD. They believed that pseudoexfoliation material might have accelerated the ischemic process of the peripapillary vessels because of vascular endothelial damage. In addition to peripapillary VD, macular VD was also reduced in PXG eyes. The authors associated this reduction with the flow disruption and ischemia due to the pseudoexfoliation material-induced endothelial damage^(19)^.

This study compared the VD in optic nerve head among POAG, PXG, and healthy eyes using OCT-A to determine the effects of pseudoexfoliation syndrome on optic nerve head vessels. We found significantly decreased VD in all regions in both POAG and PXG Groups compared with that in the control Group. In addition, the PXG Group showed significantly lower mean VD than the POAG Group in the whole image as well as the peripapillary, inside disc, and other five sectors. Moreover, the average RNFL thickness positively correlated with VD in all peripapillary sectors in the POAG and PXG Groups, unlike in the control Group. Our findings demonstrated that ischemic and vascular changes correlated with glaucoma’s pathophysiology, particularly that of pseudoexfoliation-related glaucoma. We believed that optic nerve head microvascular changes in PXG glaucoma might be an additional risk factor of faster progression and worse prognosis than in POAG.

This cross-sectional study has some limitations. We did not evaluate the progression rate of the vascular parameters in eyes with PXG and POAG compared with healthy eyes. Moreover, OCT-A may have projected artifacts although we excluded images with artifacts and signal strength index <60. Different procedures in capturing and studying data may present different outcomes. Allegrini et al.^(20)^ reported that since various projection maps could display tiny vessels that cannot be seen in standard projection, a careful approach must be considered when comparing values. The POAG and PXG Groups were matched for age, gender and data on IOP, CCT, vertical C/D ratio, and MD. However, there were more samples in the POAG Group with mild and moderate glaucoma (47.2 vs. 46.3; 33.3% vs. 29.3%), while there were more samples in the PXG Group with severe glaucoma (24.4 vs. 19.4). Moreover, we did not equate the VD within subgroups as mild to mild, moderate to moderate, and severe to severe glaucoma grades.

In conclusion, this unique study compared vascular changes in grade-matched POAG and PXG eyes using OCT-A. By generating images of the optic nerve head’s vessels, OCT-A has opened new way to understand the effects of pseudoexfoliative materials on vascular structures and functions. In addition, it is a noninvasive and more accessible imaging technique to detect microvascular deterioration. Future prospective studies assessing this novel technique’s roles in screening glaucoma progression and defining microvascular prognostic factors are needed.
